# Neuromotor Dynamics of Human Locomotion in Challenging Settings

**DOI:** 10.1016/j.isci.2019.100796

**Published:** 2019-12-24

**Authors:** Alessandro Santuz, Leon Brüll, Antonis Ekizos, Arno Schroll, Nils Eckardt, Armin Kibele, Michael Schwenk, Adamantios Arampatzis

**Affiliations:** 1 Department of Training and Movement Sciences, Humboldt-Universität zu Berlin, 10115 Berlin, Germany; 2 Berlin School of Movement Science, Humboldt-Universität zu Berlin, 10115 Berlin, Germany; 3 Atlantic Mobility Action Project, Brain Repair Centre, Department of Medical Neuroscience, Dalhousie University, Halifax, Nova Scotia B3H 4R2, Canada; 4 Network Aging Research, Heidelberg University, 69117 Heidelberg, Germany; 5 Department of Training and Movement Science, Institute for Sport and Sports Science, University of Kassel, 34125 Kassel, Germany; 6 Department of Sport and Movement Science, Institute of Sport Science, Carl von Ossietzky University of Oldenburg, 26129 Oldenburg, Germany; 7 Institute of Sports and Sports Sciences, Heidelberg University, 69117 Heidelberg, Germany

**Keywords:** Behavioral Neuroscience, Biological Sciences, Neuroscience

## Abstract

Is the control of movement less stable when we walk or run in challenging settings? Intuitively, one might answer that it is, given that challenging locomotion externally (e.g., rough terrain) or internally (e.g., age-related impairments) makes our movements more unstable. Here, we investigated how young and old humans synergistically activate muscles during locomotion when different perturbation levels are introduced. Of these control signals, called *muscle synergies*, we analyzed the local stability and the complexity (or irregularity) over time. Surprisingly, we found that perturbations force the central nervous system to produce muscle activation patterns that are less unstable and less complex. These outcomes show that robust locomotion control in challenging settings is achieved by producing less complex control signals that are more stable over time, whereas easier tasks allow for more unstable and irregular control.

## Highlights

• We examined the dynamics of motor control of locomotion in challenging settings
• We extracted muscle synergies (motor modules and primitives) from electromyography
• The dynamics of the time-dependent motor primitives were modified by perturbations
• Primitives were wider, less unstable, and complex in the presence of perturbations

## Graphical Abstract

**Figure:**
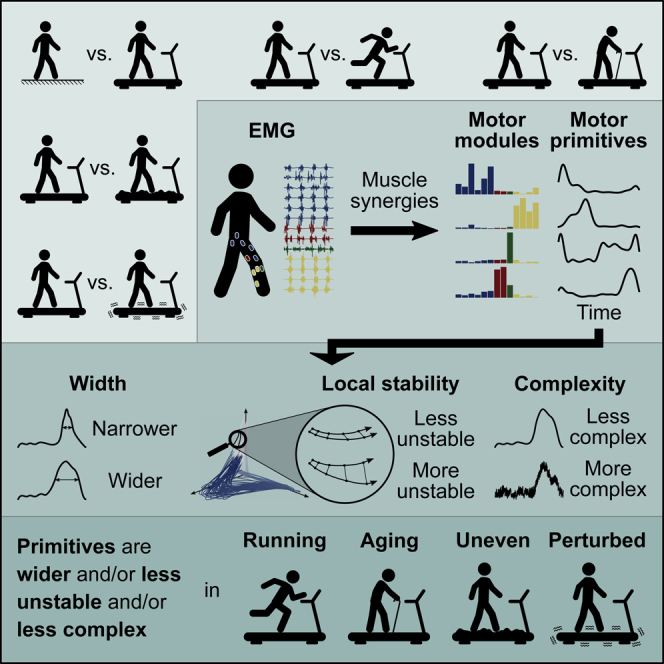

## Introduction

The central nervous system (CNS), as the fundamental nonlinear component of the majority of animals, contains both deterministic and stochastic elements (Faisal et al., 2008, Rabinovich and Abarbanel, 1998). In behaviors such as locomotion, small variations in the initial conditions might generate big variations in the evolution of the system (Lorenz, 1963). Noise affects neural control signals by adding stochastic (i.e., random) disturbances in a signal-dependent manner: if the magnitude of the control signal increases, noise levels increase as well (Harris and Wolpert, 1998). To organize robust movement patterns, the CNS must handle deterministic and stochastic variables (Faisal et al., 2008, Rabinovich and Abarbanel, 1998). Defining robustness as the ability to cope with perturbations (Santuz et al., 2018a), it follows that biological systems can manage to maintain function despite disturbances only through robust control (Kitano, 2004, Meghdadi, 2004, Shinar and Feinberg, 2010). Assessing the local stability, which is the sensitivity to infinitesimally small perturbations (Lorenz, 1963), of control signals could give us an idea of the strategies adopted by the CNS to cope with disruptions, whether they are internal (e.g., aging or disease) or external (e.g., environmental, such as changes in the morphology of terrain). This is a different kind of stability when compared with the global one. Humans are *locally* unstable due to the step-to-step variability of their movements but are at the same time *globally* stable (i.e., the variations remain within the basin of attraction) if they manage to locomote without major disruptions (Ali and Menzinger, 1999, Dingwell and Hyun, 2007, Wisse et al., 2005).

With this study, we propose an innovative approach to describe the local dynamic stability (Bradley and Kantz, 2015, Dingwell and Cusumano, 2000, Lorenz, 1963) of human motor control applied to locomotion. We started from a crucial question: how is the local stability of control signals associated to robust motor output? The answer might give important insight into the neural mechanisms necessary for the robust control of vertebrate locomotion.

Despite its nonlinear behavior, the output of the CNS can be reasonably described and modeled by means of linear approximations (Bizzi et al., 2008). The overwhelming amount of degrees of freedom available to vertebrates for accomplishing any kind of movement is defined by the vast number of muscles and joints. Yet, the CNS manages to overcome complexity, possibly through the orchestrated activation of functionally related muscle groups, rather than through muscle-specific commands (Bernstein, 1967, Bizzi et al., 1991). These common activation patterns, called *muscle synergies*, might be used by the CNS for simplifying the motor control problem by reducing its dimensionality (Bizzi et al., 2008). Usually extracted from electromyographic (EMG) data via linear machine learning approaches such as the non-negative matrix factorization (NMF), muscle synergies have been increasingly employed in the past two decades for providing indirect evidence of a simplified, modular control of movement in humans and other vertebrates (Giszter, 2015, Lacquaniti et al., 2012, Ting et al., 2015, Tresch et al., 1999).

We quantified the local dynamic stability of motor primitives (i.e., the temporal components of muscle synergies) in different locomotor tasks and settings by means of the short-term maximum Lyapunov exponents (sMLE), a metric used to describe the rate of separation of infinitesimally close trajectories (Rosenstein et al., 1993). Moreover, we used the Higuchi's fractal dimension (HFD) to evaluate the complexity of motor primitives, considered as self-similar time series (Higuchi, 1988). Namely, we considered what happens in the space of muscle synergies when humans switch from walking to running, from overground to treadmill locomotion, from unperturbed to perturbed locomotion, and in aging. We chose these conditions to compare different challenges or constraints to locomotion: running allows less time for organizing coordinated movements than walking (Ekizos et al., 2018); externally perturbed locomotion is more challenging than unperturbed locomotion due to the increased mechanical and physiological limits imposed by the higher environmental complexity (Daley, 2016, Santuz et al., 2018a); aging is a source of internal perturbation that leads to muscle weakness and loss of fine neural control at various levels (e.g., CNS, proprioceptive, etc.) (Monaco et al., 2010, Nutt et al., 1993). Recently, we and others proposed that the width of motor primitives increases to ensure robust control in the presence of internal and external perturbations (Cappellini et al., 2016, Martino et al., 2015, Martino et al., 2014, Santuz et al., 2019, Santuz et al., 2018a). Martino and colleagues were the first to interpret the widening of motor primitives as a compensatory mechanism adopted by the CNS to cope with the postural instability of locomotion in health and pathology (Martino et al., 2015, Martino et al., 2014). We observed this neural strategy in wild-type mice (Santuz et al., 2019) and in humans (Santuz et al., 2018a) undergoing external perturbations, but not in genetically modified mice that lacked feedback from proprioceptors (Santuz et al., 2019). Owing to these observations, we concluded that intact systems use wider (i.e., of longer duration) control signals to create an overlap between chronologically adjacent synergies to regulate motor function (Santuz et al., 2018a). Our conclusions, however, did not include any information about the local stability and complexity of the control signals, eventually limiting the understanding of the adaptive processes needed to cope with perturbations.

With the present analysis of human data, we discovered that less unstable and less complex motor primitives are associated with more challenging settings, whereas easier tasks allow for more unstable and more complex control. Our findings provide insight into how our CNS might control repetitive, highly stereotyped movements like locomotion, in the presence and absence of perturbations. Moreover, these results add interesting aspects to the definition of robust motor control that we and others (Aoi et al., 2019, Santuz et al., 2018a) gave in the past, integrating the concepts of local stability and complexity with the width of control signals.

## Results

### Muscle Synergies

**Figure 1:**
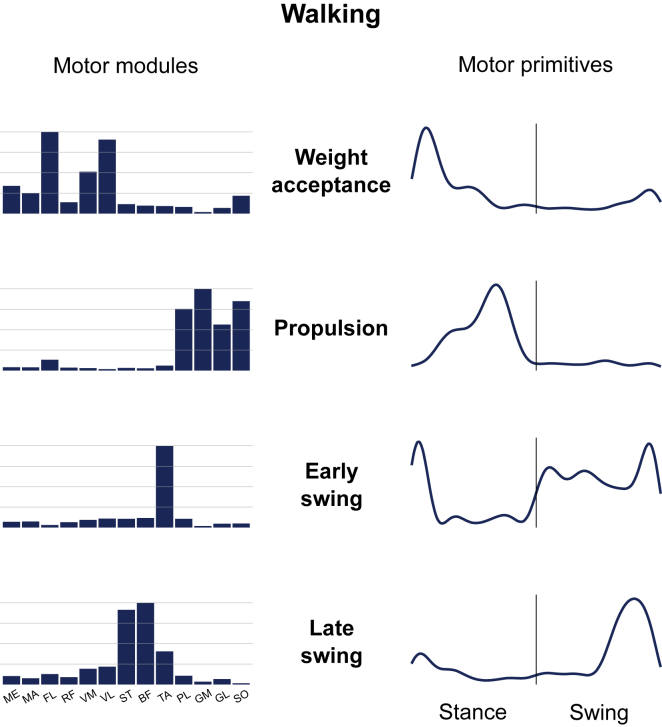
Muscle Synergies for Human Walking Exemplary motor modules and motor primitives of the four fundamental synergies for human walking (average of even-surface trials of the experimental setup E2). The motor modules are presented on a normalized y axis base. For the motor primitives, the x axis full scale represents the averaged gait cycle (with stance and swing normalized to the same amount of points and divided by a vertical line) and the y axis represents the normalized amplitude. Muscle abbreviations: ME, gluteus medius; MA, gluteus maximus; FL, tensor fasciæ latæ; RF, rectus femoris; VM, vastus medialis; VL, vastus lateralis; ST, semitendinosus; BF, biceps femoris; TA, tibialis anterior; PL, peroneus longus; GM, gastrocnemius medialis; GL, gastrocnemius lateralis; SO, soleus.

Muscle synergies for human locomotion have been extensively discussed and reported in the past (Cappellini et al., 2006, Ivanenko et al., 2004, Lacquaniti et al., 2012, Martino et al., 2015, Santuz et al., 2018a, Santuz et al., 2018b, Santuz et al., 2017a, Santuz et al., 2017b). Figure 1 shows a typical output for walking, where four fundamental synergies describe as many phases of the gait cycle. In human locomotion, the first synergy functionally refers to the body weight acceptance, with a major involvement of knee extensors and glutei. The second synergy describes the propulsion phase, to which the plantarflexors mainly contribute. The third synergy identifies the early swing, showing the involvement of foot dorsiflexors. The fourth and last synergy reflects the late swing and the landing preparation, highlighting the relevant influence of knee flexors and foot dorsiflexors.

Details about the participants are reported in the Supplemental Information. Briefly, the first group (G1) of young participants was assigned to the first experimental protocol (E1) consisting of walking and running overground and on a treadmill. The second group (G2) was assigned to the second experimental protocol (E2) consisting of walking and running on one standard and one uneven-surface (Figure S1, Video S1) treadmill. The last two groups (G3, young and G4, old) were assigned to the third and last protocol (E3) consisting of walking on a treadmill (Video S1) providing mediolateral and anteroposterior perturbations.

Video S1. Treadmills for Perturbed Locomotion Used in This Study Described in the Methods This video related to the results of Figures 3 and 4 shows the typical setup of the two treadmills used for introducing external perturbations during locomotion. The first treadmill is equipped with an uneven-surface belt. The second one can provide sudden accelerations of the belt and displacements of the platform to induce anteroposterior and mediolateral perturbations, respectively.

The minimum number of synergies that best accounted for the EMG data variance (i.e., the factorization rank) of E1 was 4.6 ± 0.5 (G1, overground walking), 4.6 ± 0.6 (G1, treadmill walking), 4.2 ± 0.7 (G1, overground running), and 4.6 ± 0.7 (treadmill running), with no significant main effects of locomotion type or condition. In E2, the values were 4.7 ± 0.7 (G2, even-surface walking), 5.1 ± 0.6 (G2, uneven-surface walking), 4.2 ± 0.6 (G2, even-surface running), and 4.6 ± 0.6 (G2, uneven-surface running), with running showing significantly less synergies than walking (p = 0.005) and no statistically significant effect of surface. Finally, in E3 the factorization ranks were 4.5 ± 0.6 (G3, unperturbed walking, young), 4.9 ± 0.5 (G3, perturbed walking, young), 4.5 ± 0.6 (G4, unperturbed walking, old), and 4.6 ± 0.5 (G4, perturbed walking, old), with perturbed walking in the young showing significantly more synergies (p < 0.001).

### Gait Cycle Parameters

The average stance and swing times together with the cadence are reported in Table 1. A main effect of speed (walking compared with running) was found in E1 and E2 for all parameters (p < 0.001) except for the swing time in E2 (p = 0.583). Treadmill, when compared with overground locomotion, made swing times decrease (p = 0.020). External perturbations (E2 and E3) influenced the stance, swing, and cadence in E3 (p < 0.001). Age played a significant role in reducing the swing times in older adults (p < 0.001). All the other comparisons were statistically (p > 0.05) not significant, and there were no interaction effects (p > 0.05).

**Table 1:** Gait Spatiotemporal Parameters

Experiment	Condition 1	Condition 2	Stance (ms)	Swing (ms)	Cadence (steps/min)
E1	Walking	Overground	689 ± 41	393 ± 26	111 ± 5
		Treadmill	674 ± 41	382 ± 30	114 ± 6
	Running	Overground	289 ± 27	473 ± 42	158 ± 11
		Treadmill	293 ± 27	449 ± 50	162 ± 12
E2	Walking	Even surface	674 ± 37	431 ± 29	109 ± 5
		Uneven surface	668 ± 49	437 ± 33	109 ± 7
	Running	Even surface	353 ± 50	424 ± 54	155 ± 7
		Uneven surface	324 ± 41	433 ± 53	159 ± 10
E3	Young	Normal walking	697 ± 32	403 ± 22	109 ± 4
		Perturbed walking	645 ± 28	377 ± 19	118 ± 5
	Old	Normal walking	693 ± 53	383 ± 23	111 ± 7
		Perturbed walking	645 ± 51	353 ± 26	121 ± 8

Stance and swing times together with the cadence are reported for the three experimental setups (values ± standard deviation).

### Perturbations Make Motor Primitives Less Unstable, Less Complex, and Fuzzier

**Figure 2:**
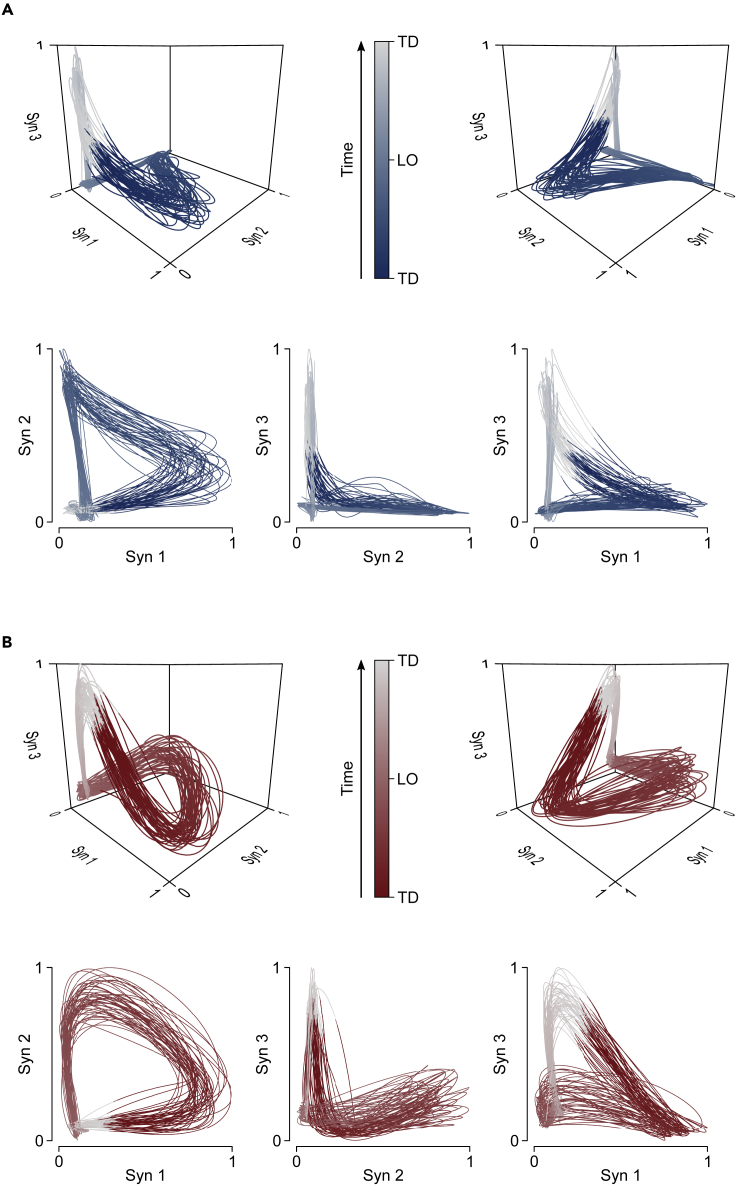
Motor Primitive Trajectories in Their Own Space (A and B) Representative data showing the filtered trajectories of motor primitives when the number of synergies (Syn) is equal to three. (A) Blue curves refer to an unperturbed walking trial recorded from a young participant. (B) Red curves refer to an unperturbed running trial recorded from a young participant. Trajectories are color coded from touchdown (TD, dark blue or red), to lift-off (LO, light blue or red), to the next TD (white). The amplitude of motor primitives is normalized to the maximum value of each trial for better visualization.

**Figure 3:**
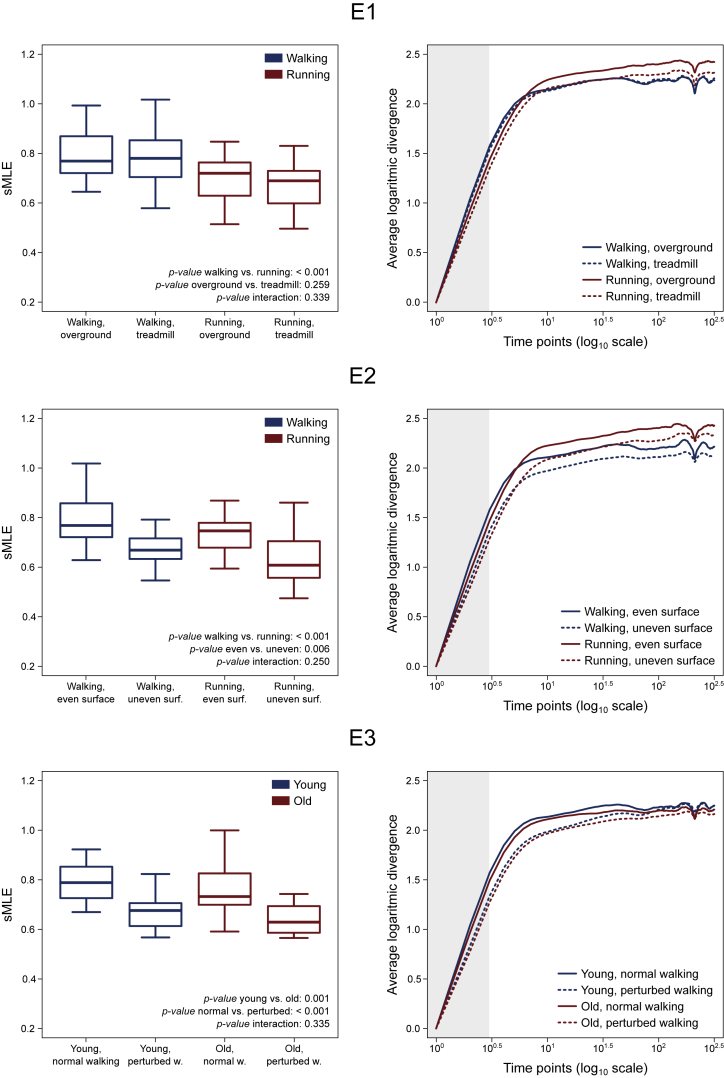
Short-Term Maximum Lyapunov Exponents of Motor Primitives Boxplots and curves describing the short-term maximum Lyapunov exponents (sMLE) and the average logarithmic divergence curves for the three experimental setups (E1, walking and running, overground and treadmill; E2, walking and running, even and uneven surface; E3, young and old, unperturbed and perturbed walking). The minimum value was subtracted from each curve for improving the visualization. The actual vertical intercept was negative and different for all curves (Figure S3). The shaded area represents the portion considered for calculating the slope. Time is presented in log_10_ scale to highlight the curve slopes. Lower sMLE imply less locally unstable motor primitives.

**Figure 4:**
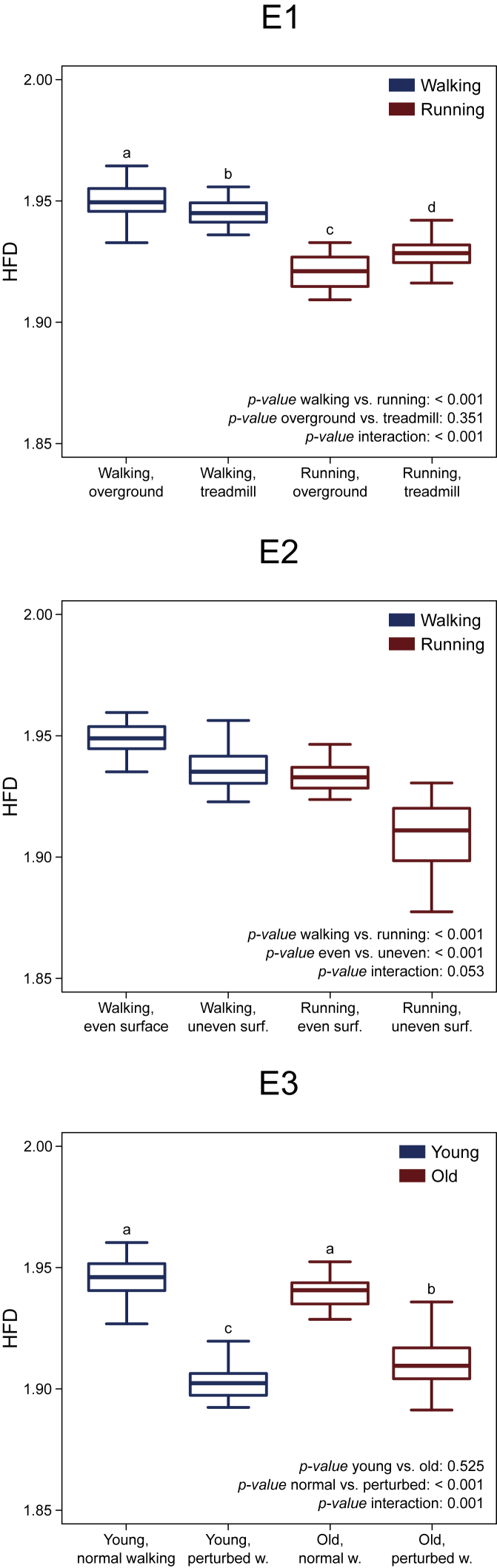
Higuchi's Fractal Dimension of Motor Primitives Boxplots describing the average Higuchi's fractal dimension (HFD) of motor primitives for the three experimental setups (E1, walking and running, overground and treadmill; E2, walking and running, even and uneven surface; E3, young and old, unperturbed and perturbed walking). Values sharing the same letter are not to be considered significantly different (results of the post-hoc analysis, where relevant).

We analyzed motor primitives in their own space, the dimension of which was equal to the trial-specific number of synergies. Two representative trials factorized into three synergies each are plotted in three-dimensional graphs in Figure 2. We used the three-dimensional example because a space in more than three dimensions would be difficult to represent. We found that motor primitives were less unstable (i.e., show lower sMLE) in (1) running compared with walking, (2) perturbed compared with unperturbed locomotion, and (3) old compared with young participants (Figures 3 and S3). Motor primitives did not show any difference in the sMLE when comparing overground with treadmill locomotion. The HFD of motor primitives are reported in Figure 4. In summary, we found that motor primitives were less complex (i.e., less irregular or with a lower fractal dimension) in (1) running compared with walking, (2) treadmill compared with overground walking, (3) overground compared with treadmill running, and (4) perturbed compared with unperturbed locomotion with older adults showing a smaller decrease in complexity than young when transitioning from unperturbed to perturbed walking (Figure 4).

In Table 2 we report synergy-by-synergy and for the three experimental setups, the factors that contributed to widen the motor primitives, thus increasing their temporal fuzziness. Detailed boxplots are available in Figure S2. Our findings show that a widening of the motor primitives, measured with the full width at half maximum (FWHM), can be observed in (1) running compared with walking, (2) perturbed compared with unperturbed locomotion, and (3) old compared with young participants (Table 2).

**Table 2:** Widening of Motor Primitives

Experiment	Synergy			p Value
E1	Weight acceptance	Walking	Running	<0.001
	Propulsion	Walking	Running	<0.001
	Early swing	Walking	Running	0.013
	Late swing	–	–	–
E2	Weight acceptance	Walking	Running	<0.001
	Propulsion	Walking	Running	<0.001
	Early swing	Walking	Running	<0.001
		Unperturbed	Perturbed	0.005
	Late swing	Walking	Running	0.001
		Unperturbed	Perturbed	<0.001
E3	Weight acceptance	Young	Old	<0.001
		Unperturbed	Perturbed	0.037
	Propulsion	Young	Old	<0.001
	Early swing	Young	Old	<0.001
		Unperturbed	Perturbed	0.042
	Late swing	Young	Old	<0.001
		Unperturbed	Perturbed	0.002

Summary of the conditions that had an effect on the full width at half maximum of the motor primitives extracted from the data of the three experimental setups (E1, walking and running, overground and treadmill; E2, walking and running, even and uneven surface; E3, young and old, unperturbed and perturbed walking). Motor primitives are the temporal coefficients of the four fundamental synergies for locomotion. Detailed boxplots are available in Figure S2.

## Discussion

Historically, the sMLE have been used to give information about the behavior of chaotic dynamical systems (Dingwell and Cusumano, 2000, Ekizos et al., 2018, Ekizos et al., 2017, Kibushi et al., 2018, Santuz et al., 2018a). In this study, we described the local stability and complexity of modular motor control in humans by calculating the sMLE and HFD of motor primitives (i.e., the time-dependent coefficients of muscle synergies) during locomotion (walking and running) overground and on a treadmill, with or without external perturbations and in aging. Our results show lower local instability (i.e., lower sMLE), lower complexity (i.e., lower HFD), and longer basic activation patterns (i.e., higher FWHM) associated with aging, external perturbations, and the switch from walking to running.

We proposed an innovative and simple approach to describe the behavior over time of neural system modularity, with an eye on increasing the reproducibility of results (all the data and code are available at Zenodo, https://doi.org/10.5281/zenodo.2669485). Typically, the sMLE are calculated from data expanded in the state space, which is a set of all the possible states of a system at any given time (Lorenz, 1963, Packard et al., 1980, Rabinovich and Abarbanel, 1998). The main assumption underlying our method is that the analysis must be conducted in the muscle synergies space, with its own dimension that is equal to the factorization rank (i.e., the minimum number of synergies necessary to sufficiently reconstruct the original EMG signals). By doing so, we did not model the whole system dynamics, but focused on the modular behavior of the CNS. In this assumption lies also the high reproducibility of our approach, because this simplification of the calculations avoids two well-known weaknesses (Bradley and Kantz, 2015) of the classical approach: the choice of time delay, which is not needed here, and state space dimension, which is calculated by NMF, for delay embedding.

HFD as a measure of irregularity or complexity has been proposed in 1988 (Higuchi, 1988) and recently rediscovered by neurophysiologists, especially for the study of electroencephalographic patterns (Kesić and Spasić, 2016, Smits et al., 2016, Zappasodi et al., 2014). Fractal time series repeat themselves at various scales, showing similar features independently on the spatial and temporal resolution we use to look at them (Higuchi, 1988, Kesić and Spasić, 2016, Smits et al., 2016, Theiler, 1990). The HFD of a monodimensional time series is a number between 1 and 2, with higher values denoting higher complexity of the signal (Smits et al., 2016). It has recently been shown that complexity in brain activity, measured by HFD, increases with maturation only to decline with aging and pathology (Kesić and Spasić, 2016, Smits et al., 2016, Zappasodi et al., 2014). Here, we show that the complexity of motor primitives is associated with external perturbations. Specifically, HFD decreases (i.e., complexity decreases) when locomotion is challenged by external perturbations in both young and older adults, even though older adults did not modulate complexity as much as the young during perturbed walking. In addition, we showed that running presented less complex motor primitives than walking, but treadmill and overground locomotion shared similar complexity values. Taken together, these results support the reduced sMLE values associated with perturbations, indicating the need for a simplification of motor control when locomotion is challenged.

In the past, we used the FWHM of motor primitives as a measure of motor control's robustness (Santuz et al., 2018a). Our conclusion was that wider (i.e., timewise longer active) primitives indicate more robust control (Santuz et al., 2018a). We reasoned that the overlap of chronologically adjacent synergies increased the fuzziness (Gentili, 2018, Meghdadi, 2004) of temporal boundaries allowing for easier shifts between one synergy (or gait phase) to the other (Santuz et al., 2018a), a conclusion that fits the optimal feedback control theory (Scott, 2004, Tuthill and Azim, 2018). For the CNS, this solution must come at a cost: the reduction of accuracy or, as others called it, optimality (Meghdadi, 2004) or efficiency (Pryluk et al., 2019). For instance, it has been recently found that human neurons allow less vocabulary overlap than monkey's, showing a trade-off between accuracy (complex human feature) and robustness (basic, typical of non-human primates) across species (Pryluk et al., 2019). In this study we confirmed a widening of motor primitives in those conditions that were more challenging than their equivalent baseline and in aging. Specifically, we considered running as a more challenging locomotion type than walking (Ekizos et al., 2018), treadmill as more challenging than overground locomotion (Dingwell and Cusumano, 2000), and perturbed locomotion as more challenging than unperturbed (Santuz et al., 2018a). We found an effect of external perturbations and aging on the widening of motor primitives.

However, we discovered that aging and the more challenging locomotion conditions not only imply wider primitives but also different local stability and complexity of neural control. We calculated lower sMLE (i.e., lower local instability) and lower HFD (i.e., lower complexity) in running compared with walking and in perturbed compared with unperturbed locomotion. Moreover, sMLE were lower in old compared with young adults. These outcomes indicate that the robustness of motor control is achieved not only by allowing motor primitives to be wider and fuzzier but also by making them less locally unstable and less complex. We recently found that the classical calculation of sMLE from kinematic data (e.g., by considering the trajectories of specific body landmarks recorded via motion capture) shows increased local instability in the presence of perturbations in both humans (Ekizos et al., 2018, Ekizos et al., 2017, Santuz et al., 2018a) and mice (Santuz et al., 2019). Our interpretation of this apparent discordance lies in the results of the present study. The sMLE calculation by means of state space reconstruction acts as a representation of the human locomotor system as a whole (Dingwell and Cusumano, 2000). Thus, increased positive sMLE mean higher sensitivity of the entire dynamical system to infinitesimal perturbations (Dingwell and Cusumano, 2000). The analysis we propose, though, aims to describe the CNS as a subsystem for the control of the main system's motion. This rationale tells us that the two descriptions are intrinsically different, possibly because they describe different portions of the “human being” as a dynamical system. From this perspective, it is not surprising that two different approaches give opposite results. In fact, the lower local instability and complexity of motor primitives might describe a strategy employed by the CNS to maintain acceptable levels of functionality when challenges are added globally to locomotion. The analysis of Lyapunov exponents has been used on EMG data as well (Kang and Dingwell, 2009). In that work, the authors found greater local dynamic instability in old compared with young adults. However, the authors only considered four muscles and made use of an eight-dimensional state space containing the four muscle activations and their time derivatives. These choices unfortunately made the work of Kang and Dingwell not directly comparable to ours.

In conclusion, our analysis of neuromotor dynamics reveals that fuzzier, less unstable, and less complex muscle activation patterns are generated by the CNS in the presence of challenging conditions to cope with perturbations (Santuz et al., 2018a). The instability and complexity of neural control decrease when movement is challenged, ensuring robust locomotion control across a variety of settings.

### Limitations of the Study

We cannot exclude (and can in fact expect) that the human system is both deterministic and stochastic. This important observation implies that our outcomes, especially those concerning the sMLE, might be influenced by both the deterministic properties of the system as well as the dynamical and measurement noise (Kantz and Schreiber, 2004). Moreover, the interpretation of the word “stability” itself can be a source of controversy, for instance, if no difference is made between *local* and *orbital* or even *global* stability (Dingwell and Hyun, 2007). In fact, when Lyapunov exponents are used together with other approaches, such as that of the maximum Floquet multipliers, the outcomes can not only be different but also opposite, for instance, with one method indicating local instability and the other indicating orbital stability (Dingwell and Hyun, 2007). To reduce these and other similar inconsistencies, we believe that future scientific endeavors should focus on finding the biological nature of metrics like the sMLE, rather than remain on the descriptive and/or speculative side.

## Methods

All methods can be found in the accompanying Transparent Methods supplemental file.

## Supplemental Information

Document S1. Transparent Methods and Figures S1–S3

## Footnotes

Supplemental Information can be found online at https://doi.org/10.1016/j.isci.2019.100796.
