# Supplementary material for: Neuromotor Dynamics of Human Locomotion in Challenging Settings
Source: iScience. 2019 Dec 24;23(1):100796. doi: 10.1016/j.isci.2019.100796 (PMC6971393; doi:10.1016/j.isci.2019.100796)
Supplement: Document S1. Transparent Methods and Figures S1–S3 [file mmc1.pdf]

iScience, Volume *23*

Supplemental Information

Neuromotor Dynamics of Human

Locomotion in Challenging Settings

Alessandro Santuz, Leon Brüll, Antonis Ekizos, Arno Schroll, Nils Eckardt, Armin Kibele, Michael Schwenk, and Adamantios Arampatzis

Neuromotor dynamics of human locomotion in challenging settings

Supplemental Information file

Transparent Methods (part of the Supplemental Information)

This study was reviewed and approved by the Ethics Committees of the Humboldt-Universität zu Berlin, Kassel University and Heidelberg University. All the participants gave written informed consent for the experimental procedure, in accordance with the Declaration of Helsinki.

Experimental protocols

For the three experimental protocols we recruited 86 healthy volunteers and divided them into four groups. The first group of 30 (henceforth G1, 15 males and 15 females, height 173 ± 10 cm, body mass 68 ± 12 kg, age 27 ± 5 years, means ± standard deviation) was assigned to the first experimental protocol (E1). The second group of 18 (G2, 11 males and 7 females, height 176 ± 7 cm, body mass 71 ± 13 kg, age 24 ± 3 years) was assigned to the second experimental protocol (E2). The last two groups where assigned to the third and last protocol (E3): one group of young (G3, 7 males and 12 females, height 171 ± 6 cm, body mass 65 ± 9 kg, age 27 ± 3 years) and one of older adults (G4, 5 males and 14 females, height 169 ± 8 cm, body mass 71 ± 12 kg, age 72 ± 6 years). All the participants completed a self-selected warm-up running on a treadmill, typically lasting between 3 and 5 min (Santuz et al., 2018b, 2016). After being instructed about the protocol, they completed a different set of measurements, depending on the protocol they were assigned to.

The experimental protocol E1 consisted of walking (at 1.40 m/s) and running (at 2.80 m/s) overground and on a treadmill (mercury, H-p-cosmos Sports & Medical GmbH, Nussdorf,

Germany). The speeds were chosen as the commonly reported average comfortable locomotion speeds (Santuz et al., 2016, 2017a). For the overground trials, we used a light-barrier system to control the speeds (average values of 1.40 ± 0.03 and 2.80 ± 0.04 m/s) in two consecutive sectors of 3 m each.

The experimental protocol E2 consisted of walking (1.10 m/s for females, 1.20 m/s for males) and running (2.00 m/s for females, 2.20 m/s for males) on one standard (Laufergotest, Erich Jäger, Würzburg, Germany) and one uneven-surface (Woodway®, Weil am Rhein, Germany, Fig. S1, Movie S1) treadmill (Santuz et al., 2018a). The uneven-surface treadmill's belt consisted of terrasensa® classic modules (Sensa® by Huebner, Kassel, Germany). The speeds were chosen after a pilot study in which we estimated the average comfortable locomotion speed on the uneven-surface treadmill for males and females separately. Part of the data from this experimental protocol was previously reported (Santuz et al., 2018a).

The experimental protocol E3 consisted of walking (1.20 m/s for the group of young adults G3, 1.10 m/s for the group of old adults G4) on a treadmill (BalanceTutor™, MediTouch LTD, Netanya, Israel, Movie S1) that could provide mediolateral (through sudden displacement of the belt-supporting platform) and anteroposterior (through rapid acceleration of the belt) perturbations. The speeds were chosen after a pilot study in which we estimated the average comfortable walking speed under perturbed conditions for young and old adults separately. Both the perturbed and unperturbed trials lasted six minutes. The perturbed trials began with ~15 s of unperturbed locomotion. Afterwards, the participants were informed about the beginning of perturbations, which were delivered randomly (left or right mediolateral displacement or acceleration) every ~3 s (G3: 3.786 ± 0.986 s; G4: 3.072 ± 0.434 sec) at unspecified phases of the gait cycle. The interval between perturbations was a function of perturbation intensity (e.g.

the larger the displacement of the platform, the longer the time needed to reset the controls and start a new perturbation). Perturbation intensities were set in the proprietary software on a scale from 1 to 30. Mediolateral perturbations were set at an intensity of 15 for G3 and 10 for G4, while the accelerations were set at 12 for G3 and 8 for G4. Intensities were chosen after a pilot study in which we defined the “most challenging intensity before failure” for young and old adults separately.

The protocols E2 and E3 both included external perturbations to locomotion. However, the timing (continuous in E2 and every 3 s in E3) and mechanics (uneven surface in E2 and displacement of the treadmill’s belt in E3) where of different nature. We chose two perturbation paradigms to allow for generalization of the outcomes. During the trials, the participants of both E2 and E3 were instructed to keep looking at a fixed spot in front of them and avoid looking at the treadmill’s belt.

<u>EMG recordings</u>

Independently on the experimental protocol, the muscle activity of the following 13 ipsilateral (right side) muscles was recorded: gluteus medius (ME), gluteus maximus (MA), tensor fasciæ latæ (FL), rectus femoris (RF), vastus medialis (VM), vastus lateralis (VL), semitendinosus (ST), biceps femoris (long head, BF), tibialis anterior (TA), peroneus longus (PL), gastrocnemius medialis (GM), gastrocnemius lateralis (GL) and soleus (SO). The electrodes were positioned as extensively reported previously (Santuz et al., 2019, 2018b). After around 60 s habituation (Santuz et al., 2018a), we recorded two trials of 60 s for each participant with an acquisition frequency of 1 kHz (E2) or 2 kHz (E1 and E3) by means of a 16-channel wireless bipolar EMG system (E2: myon m320, myon AG, Schwarzenberg, Switzerland; E1 and E3: aktos, myolution GmbH, Ratingen, Germany). For the EMG recordings, we used foam-hydrogel electrodes with

snap connector (H124SG, Medtronic plc, Dublin, Ireland). The first 30 gait cycles of the recorded trial were considered for subsequent analysis (Santuz et al., 2018b). For the overground locomotion part of E1, due to limited length of the walkway (20 m), the participants were asked to repeat the trials 10 times for the subsequent concatenation of the data. Trials that did not match the target speed with a tolerance of ± 0.05 m/s in walking and ± 0.10 m/s in running were repeated. For our analysis, we used all the available trials, taking the average of the interesting parameter when more than one trials were available. All the recordings can be downloaded from the supplementary data set, which is accessible at Zenodo (DOI: 10.5281/zenodo.2669485).

Gait cycle breakdown

The gait cycle breakdown was obtained by the elaboration of the data acquired by a 3D accelerometer operating at 148 Hz and synchronized with the EMG system. The accelerometer was strapped to the right shoe, over the most distal portion of the second to fourth metatarsal bones. The data was low-pass filtered using a 4th order IIR Butterworth zero-phase filter with cut-off frequency of 15 Hz. For estimating touchdown, we used the modified foot contact algorithm developed by Maiwald and colleagues (Maiwald et al., 2009). For estimating lift-off, we adopted our foot acceleration and jerk algorithm (Santuz et al., 2018a). The jerk algorithm searches for the global maximum of the vertical acceleration between two consecutive touchdown events to estimate the lift-off (LOe, where the "e" stays for "estimated"). This estimation, however, does not provide an accurate identification of the lift-off and needs some refinement. To get closer to the "real" lift-off timing, a characteristic minimum in the vertical acceleration (i.e. when the jerk equals zero) of the foot is identified in a reasonably small neighborhood of the LOe. We found [LOe – 250 ms, LOe + 100 ms] for both walking and running to be the sufficiently narrow interval needed to make the initial lift-off estimation.

However, we reduced this interval to [LOe – 150 ms, LOe + 100 ms] in the presence of external perturbations (i.e. for E2 and E3). Both the approaches for the determination of touchdown and lift-off have been validated using force plate data (AMTI BP600, Advanced Mechanical Technology, Inc., Watertown, MA, USA) from 15 participants walking and running overground at six different velocities without perturbations and from the data recorded in G1 overground. We then calculated the true errors between the contact times detected via force plate and those obtained from the acceleration data and used the averages to correct the calculations. Errors were of 1.9 ms for touchdown and 13.1 ms for lift-off in walking and -4.1 ms and -13.2 ms for running.

Muscle synergies extraction

For the experimental protocol E1, the overground EMG recordings were concatenated after identification of the complete gait cycles (touchdown to touchdown of the right foot). For the other protocols, we used the 30 gait cycles per trial described above. Muscle synergies data were extracted through a custom script (R v3.6.1, R Found. for Stat. Comp.) using the classical Gaussian non-negative matrix factorization (NMF) algorithm (Lee and Seung, 1999; Santuz et al., 2017a, 2018a, 2018b). The raw EMG signals were band-pass filtered within the acquisition device (cut-off frequencies 10 and 500 Hz). Then the signals were high-pass filtered, full-wave rectified and lastly low-pass filtered using a 4th order IIR Butterworth zero-phase filter with cut-off frequencies 50 Hz (high-pass) and 20 Hz (low-pass for creating the linear envelope of the signal) as previously described (Santuz et al., 2018a). After subtracting the minimum, the amplitude of the EMG recordings obtained from the single trials was normalized to the maximum activation recorded for every individual muscle (i.e. every EMG channel was normalized to its maximum in every trial) (Santuz et al., 2019, 2018b). Each gait cycle was then

time-normalized to 200 points, assigning 100 points to the stance and 100 points to the swing phase (Santuz et al., 2019, 2018a, 2018b, 2017b). The reason for this choice is twofold (Santuz et al., 2018b). First, dividing the gait cycle into two macro-phases helps the reader understanding the temporal contribution of the different synergies, diversifying between stance and swing. Second, normalizing the duration of stance and swing to the same number of points for all participants (and for all the recorded gait cycles of each participant) makes the interpretation of the results independent from the absolute duration of the gait events. Synergies were then extracted through NMF as previously described (Santuz et al., 2018a, 2018b). For the analysis, we considered the 13 muscles described above (ME, MA, FL, RF, VM, VL, ST, BF, TA, PL, GM, GL and SO). The m = 13 time-dependent muscle activity vectors were grouped in a matrix V with dimensions m × n (m rows and n columns). The dimension n represented the number of normalized time points (i.e. 200*number of gait cycles). The matrix V was factorized using NMF so that $V \approx V_R = WH$. The new matrix V_R, reconstructed multiplying the two matrices W and H, approximates the original matrix V. The motor primitives (Dominici et al., 2011; Santuz et al., 2017a) matrix H contained the time-dependent coefficients of the factorization with dimensions r × n, where the number of rows r represents the minimum number of synergies necessary to satisfactorily reconstruct the original set of signals V. The motor modules (Gizzi et al., 2011; Santuz et al., 2017a) matrix W, with dimensions m × r, contained the time-invariant muscle weightings, which describe the relative contribution of single muscles within a specific synergy (a weight was assigned to each muscle for every synergy). H and W described the synergies necessary to accomplish the required task (i.e. walking or swimming). The update rules for W and H are presented in Equation (EQ1) and Equation (EQ2).

$$\begin{cases} H_{i+1} = H_i \dfrac{{W_i}^T V}{{W_i}^T W_i H_i} & \text{(EQ1)} \\ W_{i+1} = W_i \dfrac{V(H_{i+1})^T}{W_i H_{i+1} (H_{i+1})^T} & \text{(EQ2)} \end{cases}$$

The quality of reconstruction was assessed by measuring the coefficient of determination R^2 between the original and the reconstructed data (V and V_R, respectively). The limit of convergence for each synergy was reached when a change in the calculated R^2 was smaller than the 0.01% in the last 20 iterations (Santuz et al., 2017a) meaning that, with that amount of synergies, the signal could not be reconstructed any better. This operation was first completed by setting the number of synergies to 1. Then, it was repeated by increasing the number of synergies each time, until a maximum of 10 synergies. The number 10 was chosen to be lower than the number of muscles, since extracting a number of synergies equal to the number of measured EMG activities would not reduce the dimensionality of the data. Specifically, 10 is the rounded 75% of 13, which is the number of considered muscles (Santuz et al., 2019). For each synergy, the factorization was repeated 10 times, each time creating new randomized initial matrices W and H, in order to avoid local minima (D’Avella and Bizzi, 2005). The solution with the highest R^2 was then selected for each of the 10 synergies. To choose the minimum number of synergies required to represent the original signals, the curve of R^2 values versus synergies was fitted using a simple linear regression model, using all 10 synergies. The mean squared error (Cheung et al., 2005) between the curve and the linear interpolation was then calculated. Afterwards, the first point in the R^2-vs.-synergies curve was removed and the error between this new curve and its new linear interpolation was calculated. The operation was repeated until only two points were left on the curve or until the mean squared error fell below 10^{-4}. This was done to search for the

most linear part of the R^2-versus-synergies curve, assuming that in this section the reconstruction quality could not increase considerably when adding more synergies to the model.

Local dynamic stability of motor primitives

We assessed the local dynamic stability of motor primitives using the short-term maximum Lyapunov exponents (sMLE) (Rosenstein et al., 1993). Usually sMLE are extracted after reconstruction of the state space through delay-coordinate embedding starting from a measured one-dimensional time series (Packard et al., 1980). The state space is a set of all the possible states of a system at any given time, the variables of which might be position, velocity, temperature, color, species, voltage and many others (Lorenz, 1963; Packard et al., 1980; Rabinovich and Abarbanel, 1998). Yet, in our typical experimental setups involving complex living systems like humans, the state space is often unknown. Theoretically, the behavior of a purely chaotic dynamical system can be predicted by using only a small set of observations on its state (e.g. joint angles, or kinematics, or accelerations, etc.) without losing information on its properties (Ekizos et al., 2018; Santuz et al., 2018a). For this reason, it is common to use the recorded data to reconstruct the state space, usually by means of the ”delay embedding theorem” (Takens, 1981). Typically sMLE are then calculated from data expanded in the state space (Lorenz, 1963; Packard et al., 1980). We avoided this passage by assuming that the space we are interested into had dimension equal to the factorization rank (i.e. the minimum number of synergies necessary to sufficiently reconstruct the original EMG signals). That is, the embedding dimension was determined by the NMF. The work from Sauer and colleagues (Sauer et al., 1991) allows to use n-dimensional measurements instead of the classical state space reconstruction. For instance, if one trial was factorized by NMF into four synergies, we would calculate the sMLE of the resulting four motor primitives using four as embedding dimension. This approach has the

advantage that it does not require the estimation of a suitable delay and embedding dimension, the latter being particularly sensitive to the presence of noise, which is very likely in experimental data (Dingwell et al., 2007). The motor primitives associated to the synergies extracted using the methods described above, were analyzed as follows. Each set of motor primitives was a time series of 30 gait cycles, normalized in time as described above (100 points for the stance and 100 for the swing, for a total of n=6000 points per trial). Primitives were then scaled to have the same variance by subtracting the mean and dividing by the standard deviation in order to avoid having different dynamical ranges across the data set (Kantz and Schreiber, 2004). For every point x_i in each time series (or set of motor primitives), we searched for the nearest neighbors of the point x_i excluding the neighborhood points [x_{i-100}, x_{i+100}]. This interval was chosen in order to impose a temporal separation between the nearest neighbors (Theiler window), making sure that they were on different trajectories (or gait cycles), as previously described (Rosenstein et al., 1993). Once the algorithm found the nearest neighbors, we proceeded to calculate the logarithm of the divergence between the trajectory of each point and its nearest neighbor's and for a maximum of 300 consecutive time points. For each trial, the divergence curve was calculated as the average of all divergence curves obtained from each point in the time series and their neighbors (Rosenstein et al., 1993). We then defined sMLE as the slope of the most linear part of the divergence curve, starting from the first point. To define linearity, we imposed the R^2 between the curve and its linear interpolation to be bigger than 0.9. Across all trials for each experimental setup, we then found the minimum number of points needed to reach a linear interpolation with $R^2 > 0.9$ and used these values to recalculate the final sMLE. The minimum number of points was three in E1, E2 and E3 and this is the value we used;

the maximum was 7 (E1), 8 (E2), and 6 (E3), with average values of 4.7 ± 0.9 (E1), 4.8 ± 1.0 (E2), 4.6 ± 0.6 (E3).

Higuchi's fractal dimension of motor primitives

To assess the irregularity (or complexity) of motor primitives, we calculated the Higuchi's fractal dimension (HFD), assuming that these time series exhibit self-similarity properties (Higuchi, 1988; Kesić and Spasić, 2016; Smits et al., 2016; Theiler, 1990). Following the procedure first described by Higuchi (Higuchi, 1988), for each motor primitive *H(t)*[*H(1)*, *H(2)*, ... *H(n)*], we constructed k sets of new time series, where k is an integer interval time and $2 < k < k_{max}$:

$$H_k^{t_0}: H(t_0), H(t_0 + k), H(t_0 + 2k), \dots, H\left[t_0 + int\left(\frac{n - t_0}{k}\right)k\right] \qquad (EQ3)$$

where t_0 is the first sample at initial time. The non-Euclidean length of each curve was defined as

$$L_{t_0}(k) = \frac{1}{k}\left\{\frac{n-1}{int\left(\frac{n-t_0}{k}\right)k}\left[\sum_{i=1}^{int\left(\frac{n-t_0}{k}\right)k} |H(t_0 + ik) - H(t_0 + (i-1)k)|\right]\right\} \qquad (EQ4)$$

and for every considered k step the length of the motor primitive was defined as the average of the k sets of lengths as

$$L(k) = \frac{1}{k}\sum_{t_0=1}^{k} L_{t_0}(k) \qquad (EQ5).$$

If L(k) ∝ k^{-HFD}, then the curve is fractal with dimension HFD and this should lead the plot of log(L(k)) versus log(1/k) to fall on a straight line with slope -HFD. For each trial, the HFD of the primitives obtained by NMF were calculated separately and then averaged, so that each trial ultimately consisted of one HFD value. Since HFD increases with k_{max} until it reaches a plateau at $k_{plateau}$, we chose as k_{max} the average of the $k_{plateau}$ values obtained from all trials. In this study, $k_{max} = 237$ time points.

Width of motor primitives

We compared motor primitives by evaluating the full width at half maximum (FWHM), a metric useful to describe the duration of activation patterns (Cappellini et al., 2006; Martino et al., 2014; Santuz et al., 2019, 2018a). The FWHM was calculated cycle-by-cycle as the number of points exceeding each cycle's half maximum, after subtracting the cycle's minimum and then averaged (Martino et al., 2014). The FWHM (and just this parameter) was calculated only for the motor primitives relative to fundamental synergies. A fundamental synergy can be defined as an activation pattern whose motor primitive shows a single main peak of activation (Santuz et al., 2018a). When two or more fundamental synergies are blended into one, a combined synergy appears. Combined synergies usually constitute, in our data, 10 to 30% of the total extracted synergies. Due to the lack of consent in the literature on how to interpret them, we excluded the combined synergies from the FWHM analysis. The recognition of fundamental synergies was carried out based on a previously reported approach (Santuz et al., 2017b, 2017a), which involves the creation of a training set and the subsequent supervised clustering of similar primitives.

Statistics

To investigate the main effects on the sMLE, HFD, and FWHM of locomotion type (i.e. walking or running), condition (i.e. overground or treadmill), perturbations and age, we fitted the data using a generalized linear model with Gaussian error distribution. The homogeneity of variances was tested using the Levene's test. If the residuals were normally distributed, we carried out a two-way repeated measures ANOVA with type II sum of squares, the independent variables being: locomotion type (walking or running) and condition (overground or treadmill) in E1; locomotion type (walking or running) and condition (perturbed or unperturbed) in E2;

locomotion condition (perturbed or unperturbed) and age (young or old) in E3. If the normality assumptions on the residuals were not met, we used a robust (rank-based) ANOVA from the R package Rfit (function “raov”) (Kloke and McKean, 2012; McKean and Kloke, 2014). When an interaction of the main effects was observed, we performed a least significant difference *post-hoc* analysis with false discovery rate adjustment of the *p*-values. All the significance levels were set to $\alpha = 0.05$ and the statistical analyses were conducted using R v3.6.1 (R Found. for Stat. Comp.).

Data and code availability

In the supplementary data set accessible at Zenodo (DOI: 10.5281/zenodo.2669485) we made available: a) the metadata with anonymized participant information, b) the raw EMG, c) the touchdown and lift-off timings of the recorded limb, d) the filtered and time-normalized EMG, e) the muscle synergies extracted via NMF and f) the code to process the data, including the scripts to calculate the sMLE and HFD of motor primitives. In total, 476 trials from 86 participants are included in the supplementary data set.

The file “participant_data.dat” is available in ASCII and RData (R Found. for Stat. Comp.) format and contains:

- Code: the participant’s code
- Experiment: the experimental setup in which the participant was involved (E1 = walking and running, overground and treadmill; E2 = walking and running, even- and uneven-surface; E3 = unperturbed and perturbed walking, young and old)
- Group: the group to which the participant was assigned (see methods for the details)
- Sex: the participant’s sex (M or F)

- Speed: the speed at which the recordings were conducted in [m/s] (two values separated by a comma mean that recordings were done at two different speeds, i.e. walking and running)
- Age: the participant's age in years (participants were considered old if older than 65 years, but younger than 80)
- Height: the participant's height in [cm]
- Mass: the participant's body mass in [kg].

The files containing the gait cycle breakdown are available in RData (R Found. for Stat. Comp.) format, in the file named "CYCLE_TIMES.RData". The files are structured as data frames with 30 rows (one for each gait cycle) and two columns. The first column contains the touchdown incremental times in seconds. The second column contains the duration of each stance phase in seconds. Each trial is saved as an element of a single R list. Trials are named like "CYCLE_TIMES_P0020," where the characters "CYCLE_TIMES" indicate that the trial contains the gait cycle breakdown times and the characters "P0020" indicate the participant number (in this example the 20th). Please note that the overground trials of participants P0001 and P0009 and the second uneven-surface running trial of participant P0048 only contain 22, 27 and 23 cycles, respectively.

The files containing the raw, filtered and the normalized EMG data are available in RData (R Found. for Stat. Comp.) format, in the files named "RAW_EMG.RData" and "FILT_EMG.RData". The raw EMG files are structured as data frames with 30000 rows (one for each recorded data point) and 14 columns. The first column contains the incremental time in seconds. The remaining thirteen columns contain the raw EMG data, named with muscle abbreviations that follow those reported in the Materials and Methods section of this

Supplementary Materials file. Each trial is saved as an element of a single R list. Trials are named like “RAW_EMG_P0053_OW_02”, where the characters “RAW_EMG” indicate that the trial contains raw EMG data, the characters “P0053” indicate the participant number (in this example the 53rd), the characters “OW” indicate the locomotion type (E1: OW=overground walking, OR=overground running, TW=treadmill walking, TR=treadmill running; E2: EW=even-surface walking, ER=even-surface running, UW=uneven-surface walking, UR=uneven-surface running; E3: NW=normal walking, PW=perturbed walking), and the numbers “02” indicate the trial number (in this case the 2nd). The 10 trials per participant recorded for each overground session (i.e. 10 for walking and 10 for running) were concatenated into one. The filtered and time-normalized EMG data is named, following the same rules, like “FILT_EMG_P0053_OW_02”.

The files containing the muscle synergies extracted from the filtered and normalized EMG data are available in RData (R Found. for Stat. Comp.) format, in the files named “SYNS_H.RData” and “SYNS_W.RData”. The muscle synergies files are divided in motor primitives and motor modules and are presented as direct output of the factorization and not in any functional order. Motor primitives are data frames with 6000 rows and a number of columns equal to the number of synergies (which might differ from trial to trial) plus one. The rows contain the time-dependent coefficients (motor primitives), one column for each synergy plus the time points (columns are named e.g. “Time, Syn1, Syn2, Syn3”, where “Syn” is the abbreviation for “synergy”). Each gait cycle contains 200 data points, 100 for the stance and 100 for the swing phase which, multiplied by the 30 recorded cycles, result in 6000 data points distributed in as many rows. This output is transposed as compared to the one discussed in the methods section to improve user readability. Each set of motor primitives is saved as an element of a single R list.

Trials are named like “SYNS_H_P0012_PW_02”, where the characters “SYNS_H” indicate that the trial contains motor primitive data, the characters “P0012” indicate the participant number (in this example the 12th), the characters “PW” indicate the locomotion type (see above), and the numbers “02” indicate the trial number (in this case the 2nd). Motor modules are data frames with 13 rows (number of recorded muscles) and a number of columns equal to the number of synergies (which might differ from trial to trial). The rows, named with muscle abbreviations that follow those reported in the methods section, contain the time-independent coefficients (motor modules), one for each synergy and for each muscle. Each set of motor modules relative to one synergy is saved as an element of a single R list. Trials are named like “SYNS_W_P0082_PW_02”, where the characters “SYNS_W” indicate that the trial contains motor module data, the characters “P0082” indicate the participant number (in this example the 82nd), the characters “PW” indicate the locomotion type (see above), and the numbers “02” indicate the trial number (in this case the 2nd). Given the nature of the NMF algorithm for the extraction of muscle synergies, the supplementary data set might show non-significant differences as compared to the one used for obtaining the results of this paper.

The files containing the sMLE calculated from motor primitives are available in RData (R Found. for Stat. Comp.) format, in the file named “sMLE.RData”. sMLE results are presented in a list of lists containing, for each trial, 1) the divergences, 2) the sMLE, and 3) the value of the R^2 between the divergence curve and its linear interpolation made using the specified amount of points. The divergences are presented as one-dimensional vectors. sMLE are one number like the R^2 value. Trials are named like “sMLE_P0081_EW_01”, where the characters “sMLE” indicate that the trial contains sMLE data, the characters “P0081” indicate the participant number (in this

example the 81st), the characters "EW" indicate the locomotion type (see above), and the numbers "01" indicate the trial number (in this case the 1st).

The files containing the HFD calculated from motor primitives are available in RData (R Found. for Stat. Comp.) format, in the file named "HFD.RData". HFD results are presented in a list of lists containing, for each trial, 1) the HFD, and 2) the interval time *k* used for the calculations. HFDs are presented as one number, as are the interval times *k*. Trials are named like "HFD_P0048_TR_01", where the characters "HFD" indicate that the trial contains HFD data, the characters "P0048" indicate the participant number (in this example the 48th), the characters "TR" indicate the locomotion type (see above), and the numbers "01" indicate the trial number (in this case the 1st).

All the code used for the preprocessing of EMG data, the extraction of muscle synergies, the calculation of sMLE and HFD is available in R (R Found. for Stat. Comp.) format. Explanatory comments are profusely present throughout the scripts ("SYNS.R", which is the script to extract synergies, "fun_NMF.R", which contains the NMF function, "sMLE.R", which is the script to calculate the sMLE of motor primitives, "HFD.R", which is the script to calculate the HFD of motor primitives, "fun_sMLE.R", which contains the sMLE function and "fun_HFD.R", which contains the HFD function).

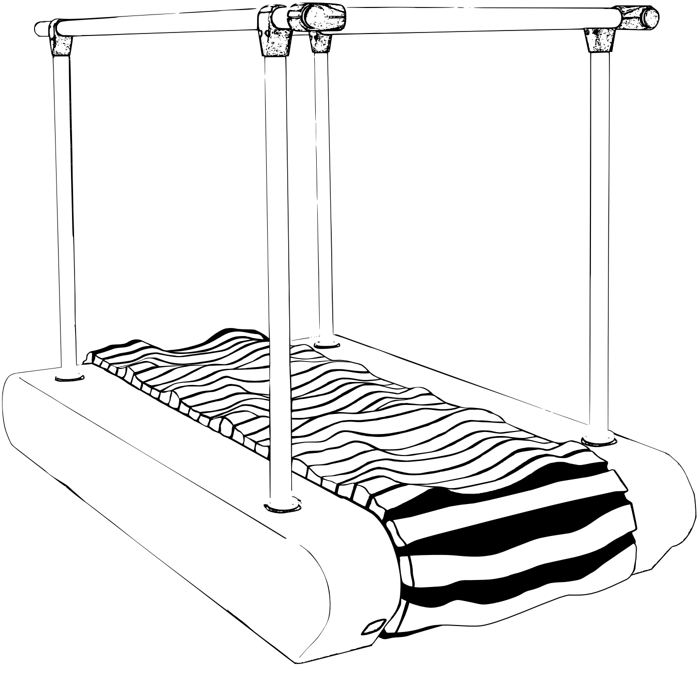

Fig. S1. Sketch of the uneven-surface treadmill used for the experimental protocol E2 described in the methods and related to Fig. 3 and Fig. 4. The belt of this treadmill was built to reproduce an uneven-terrain environment.

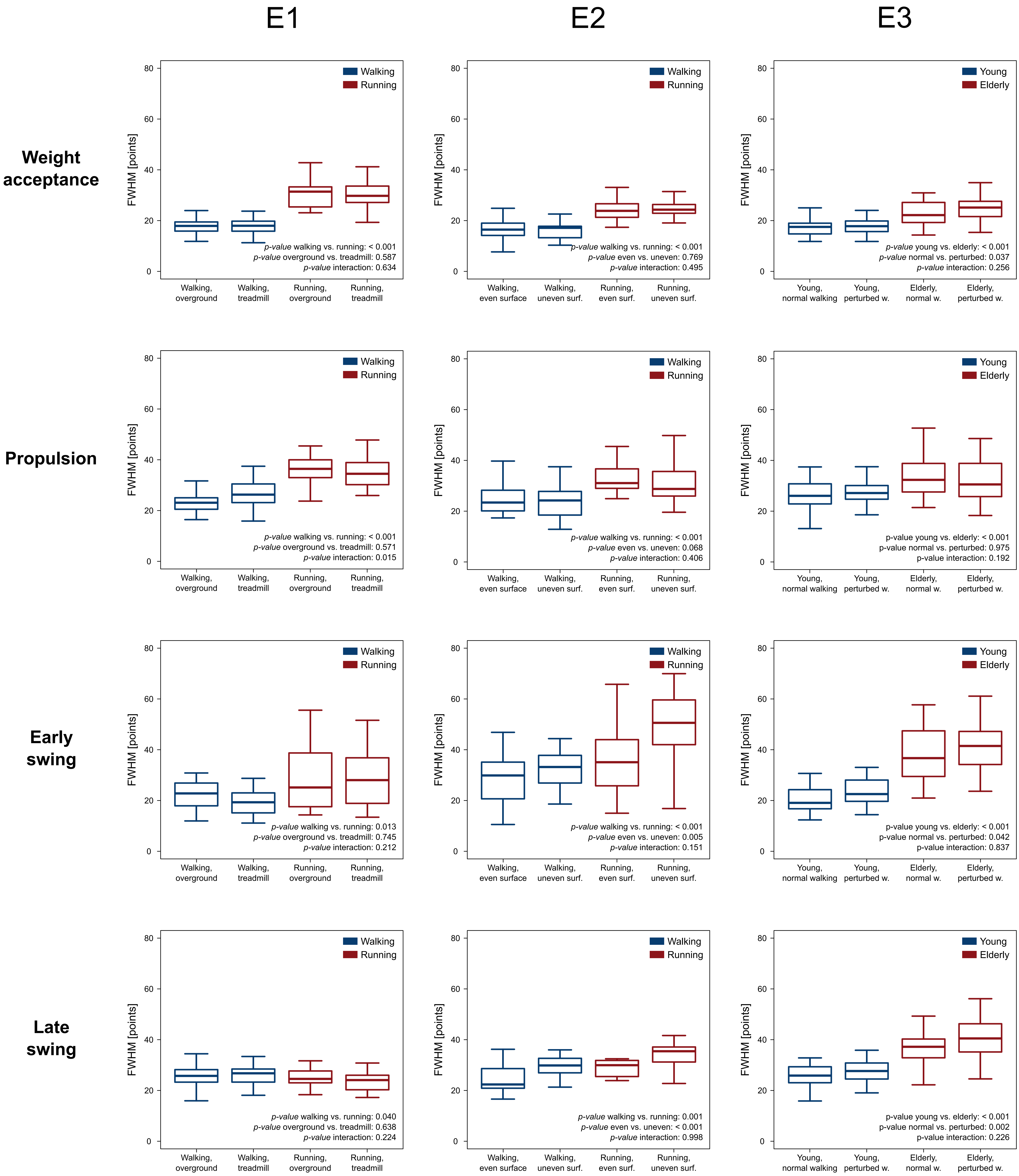
E1
E2
E3
Weight acceptance
Propulsion
Early swing
Late swing
FWHM [points]
Walking
Running
Young
Elderly
Walking, overground
Walking, treadmill
Running, overground
Running, treadmill
Walking, even surface
Walking, uneven surf.
Running, even surf.
Running, uneven surf.
Young, normal walking
Young, perturbed w.
Elderly, normal w.
Elderly, perturbed w.
p-value walking vs. running: < 0.001
p-value overground vs. treadmill: 0.587
p-value interaction: 0.634
p-value walking vs. running: < 0.001
p-value even vs. uneven: 0.769
p-value interaction: 0.495
p-value young vs. elderly: < 0.001
p-value normal vs. perturbed: 0.037
p-value interaction: 0.256
p-value walking vs. running: < 0.001
p-value overground vs. treadmill: 0.571
p-value interaction: 0.015
p-value walking vs. running: < 0.001
p-value even vs. uneven: 0.068
p-value interaction: 0.406
p-value young vs. elderly: < 0.001
p-value normal vs. perturbed: 0.975
p-value interaction: 0.192
p-value walking vs. running: 0.013
p-value overground vs. treadmill: 0.745
p-value interaction: 0.212
p-value walking vs. running: < 0.001
p-value even vs. uneven: 0.005
p-value interaction: 0.151
p-value young vs. elderly: < 0.001
p-value normal vs. perturbed: 0.042
p-value interaction: 0.837
p-value walking vs. running: 0.040
p-value overground vs. treadmill: 0.638
p-value interaction: 0.224
p-value walking vs. running: 0.001
p-value even vs. uneven: < 0.001
p-value interaction: 0.998
p-value young vs. elderly: < 0.001
p-value normal vs. perturbed: 0.002
p-value interaction: 0.226

Fig. S2. Full width at half maximum of motor primitives related to Table 2. Boxplots describing the full width at half maximum (FWHM) of the motor primitives extracted from the data of the three experimental setups (E1 = walking and running, overground and treadmill; E2 = walking and running, even- and uneven-surface; E3 = unperturbed and perturbed walking, young and old). Motor primitives are the temporal coefficients of the four fundamental synergies for locomotion. Lower FWHM imply shorter duration of activation.

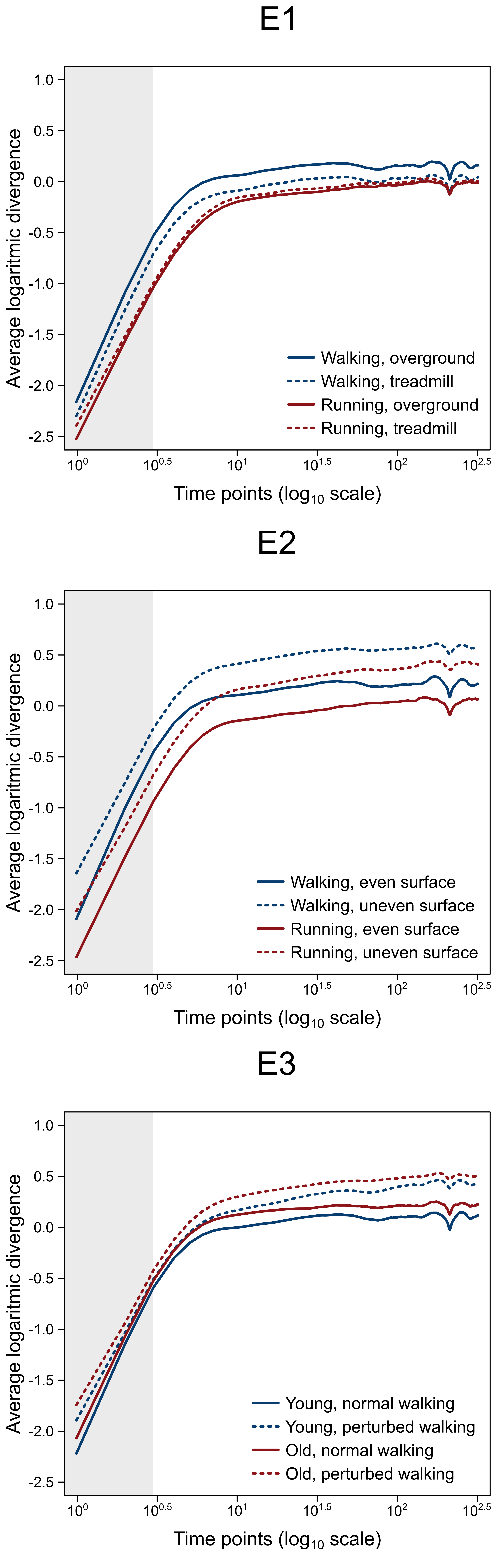
E1
Average logaritmic divergence
1.0
0.5
0.0
-0.5
-1.0
-1.5
-2.0
-2.5
Walking, overground
Walking, treadmill
Running, overground
Running, treadmill
10^0
$10^{0.5}$
10^1
$10^{1.5}$
10^2
$10^{2.5}$
Time points ($\log_{10}$ scale)
E2
Average logaritmic divergence
1.0
0.5
0.0
-0.5
-1.0
-1.5
-2.0
-2.5
Walking, even surface
Walking, uneven surface
Running, even surface
Running, uneven surface
10^0
$10^{0.5}$
10^1
$10^{1.5}$
10^2
$10^{2.5}$
Time points ($\log_{10}$ scale)
E3
Average logaritmic divergence
1.0
0.5
0.0
-0.5
-1.0
-1.5
-2.0
-2.5
Young, normal walking
Young, perturbed walking
Old, normal walking
Old, perturbed walking
10^0
$10^{0.5}$
10^1
$10^{1.5}$
10^2
$10^{2.5}$
Time points ($\log_{10}$ scale)

Fig. S3. Average logarithmic divergence curves of motor primitives with their original vertical intercept related to Fig. 3. Curves describing the average logarithmic divergence curves for the three experimental setups (E1 = walking and running, overground and treadmill; E2 = walking and running, even- and uneven-surface; E3 = unperturbed and perturbed walking, young and old). The shaded area represents the portion considered for calculating the slope. Time is presented in $\log_{10}$ scale to highlight the curve slopes.

Supplemental references

Cappellini, G., Ivanenko, Y.P., Poppele, R.E., Lacquaniti, F., 2006. Motor patterns in human walking and running. J. Neurophysiol. 95, 3426–37. https://doi.org/10.1152/jn.00081.2006

Cheung, V.C.-K., D'Avella, A., Tresch, M.C., Bizzi, E., 2005. Central and sensory contributions to the activation and organization of muscle synergies during natural motor behaviors. J. Neurosci. 25, 6419–34. https://doi.org/10.1523/JNEUROSCI.4904-04.2005

D'Avella, A., Bizzi, E., 2005. Shared and specific muscle synergies in natural motor behaviors. Proc. Natl. Acad. Sci. U. S. A. 102, 3076–81. https://doi.org/10.1073/pnas.0500199102

Dingwell, J.B., Kang, H.G., Marin, L.C., 2007. The effects of sensory loss and walking speed on the orbital dynamic stability of human walking. J. Biomech. 40, 1723–30. https://doi.org/10.1016/j.jbiomech.2006.08.006

Dominici, N., Ivanenko, Y.P., Cappellini, G., D'Avella, A., Mondi, V., Cicchese, M., Fabiano, A., Silei, T., Di Paolo, A., Giannini, C., Poppele, R.E., Lacquaniti, F., 2011. Locomotor Primitives in Newborn Babies and Their Development. Science (80-.). 334, 997–999. https://doi.org/10.1126/science.1210617

Ekizos, A., Santuz, A., Schroll, A., Arampatzis, A., 2018. The Maximum Lyapunov Exponent During Walking and Running: Reliability Assessment of Different Marker-Sets. Front. Physiol. 9, 1101. https://doi.org/10.3389/fphys.2018.01101

Gizzi, L., Nielsen, J.F., Felici, F., Ivanenko, Y.P., Farina, D., 2011. Impulses of activation but not motor modules are preserved in the locomotion of subacute stroke patients. J. Neurophysiol. 106, 202–210. https://doi.org/10.1152/jn.00727.2010

Higuchi, T., 1988. Approach to an irregular time series on the basis of the fractal theory. Phys. D

Nonlinear Phenom. 31, 277–283. https://doi.org/10.1016/0167-2789(88)90081-4

Kantz, H., Schreiber, T., 2004. Nonlinear Time Series Analysis, 2nd ed. Cambridge University Press, Cambridge, UK.

Kesić, S., Spasić, S.Z., 2016. Application of Higuchi's fractal dimension from basic to clinical neurophysiology: A review. Comput. Methods Programs Biomed. 133, 55–70. https://doi.org/10.1016/j.cmpb.2016.05.014

Kloke, J.D., McKean, J.W., 2012. Rfit: Rank-based Estimation for Linear Models. R J. 4, 57–64.

Lee, D.D., Seung, H.S., 1999. Learning the parts of objects by non-negative matrix factorization. Nature 401, 788–91. https://doi.org/10.1038/44565

Lorenz, E.N., 1963. Deterministic Nonperiodic Flow. J. Atmos. Sci. 20, 130–141. https://doi.org/10.1175/1520-0469(1963)020<0130:DNF>2.0.CO;2

Maiwald, C., Sterzing, T., Mayer, T.A., Milani, T.L., 2009. Detecting foot-to-ground contact from kinematic data in running. Footwear Sci. 1, 111–118. https://doi.org/10.1080/19424280903133938

Martino, G., Ivanenko, Y.P., Serrao, M., Ranavolo, A., D'Avella, A., Draicchio, F., Conte, C., Casali, C., Lacquaniti, F., 2014. Locomotor patterns in cerebellar ataxia. J. Neurophysiol. 112, 2810–2821. https://doi.org/10.1152/jn.00275.2014

McKean, J.W., Kloke, J.D., 2014. Efficient and adaptive rank-based fits for linear models with skew-normal errors. J. Stat. Distrib. Appl. 1–18. https://doi.org/10.1186/s40488-014-0018-0

Packard, N.H., Crutchfield, J.P., Farmer, J.D., Shaw, R.S., 1980. Geometry from a Time Series. Phys. Rev. Lett. 45, 712–716. https://doi.org/10.1103/PhysRevLett.45.712

Rabinovich, M.., Abarbanel, H.D.., 1998. The role of chaos in neural systems. Neuroscience 87, 5–14. https://doi.org/10.1016/S0306-4522(98)00091-8

Rosenstein, M.T., Collins, J.J., De Luca, C.J., 1993. A practical method for calculating largest Lyapunov exponents from small data sets. Phys. D 65, 117–134. https://doi.org/10.1016/0167-2789(93)90009-P

Santuz, A., Akay, T., Mayer, W.P., Wells, T.L., Schroll, A., Arampatzis, A., 2019. Modular organization of murine locomotor pattern in the presence and absence of sensory feedback from muscle spindles. J. Physiol. 597, 3147–3165. https://doi.org/10.1113/JP277515

Santuz, A., Ekizos, A., Arampatzis, A., 2016. A Pressure Plate-Based Method for the Automatic Assessment of Foot Strike Patterns During Running. Ann. Biomed. Eng. 44, 1646–1655. https://doi.org/10.1007/s10439-015-1484-3

Santuz, A., Ekizos, A., Eckardt, N., Kibele, A., Arampatzis, A., 2018a. Challenging human locomotion: stability and modular organisation in unsteady conditions. Sci. Rep. 8, 2740. https://doi.org/10.1038/s41598-018-21018-4

Santuz, A., Ekizos, A., Janshen, L., Baltzopoulos, V., Arampatzis, A., 2017a. On the Methodological Implications of Extracting Muscle Synergies from Human Locomotion. Int. J. Neural Syst. 27, 1750007. https://doi.org/10.1142/S0129065717500071

Santuz, A., Ekizos, A., Janshen, L., Baltzopoulos, V., Arampatzis, A., 2017b. The Influence of Footwear on the Modular Organization of Running. Front. Physiol. 8, 958. https://doi.org/10.3389/fphys.2017.00958

Santuz, A., Ekizos, A., Janshen, L., Mersmann, F., Bohm, S., Baltzopoulos, V., Arampatzis, A., 2018b. Modular Control of Human Movement During Running: An Open Access Data Set.

Front. Physiol. 9, 1509. https://doi.org/10.3389/fphys.2018.01509

Sauer, T., Yorke, J.A., Casdagli, M., 1991. Embedology. J. Stat. Phys. 65, 579–616. https://doi.org/10.1007/BF01053745

Smits, F.M., Porcaro, C., Cottone, C., Cancelli, A., Rossini, P.M., Tecchio, F., 2016. Electroencephalographic Fractal Dimension in Healthy Ageing and Alzheimer's Disease. PLoS One 11, e0149587. https://doi.org/10.1371/journal.pone.0149587

Takens, F., 1981. Detecting strange attractors in turbulence, in: Rand, D., Young, L.-S. (Eds.), Dynamical Systems and Turbulence, Warwick 1980. Springer Berlin Heidelberg, Berlin, Heidelberg, pp. 366–381. https://doi.org/10.1007/BFb0091924

Theiler, J., 1990. Estimating the Fractal Dimension of Chaotic Time Series. Lincoln Lab. J. 3, 63–86. https://doi.org/10.1.1.229.3288
